# Reliability of mpMRI in diagnosing cancer prostate following intravesical BCG for bladder cancer

**DOI:** 10.1002/bco2.446

**Published:** 2024-10-15

**Authors:** Arjun Pon Avudaiappan, Pushan Prabhakar, Rachel Siretskiy, Andrew Renshaw, Ahmed Eldefrawy, Murugesan Manoharan

**Affiliations:** ^1^ Department of Urologic Oncology Surgery Miami Cancer Institute Miami Florida USA; ^2^ Herbert Wertheim College of Medicine Florida International University Miami Florida USA

**Keywords:** cancer prostate, granulomatous prostatitis, intravesical BCG, mpMRI, non‐muscle invasive bladder cancer

## Abstract

**Background:**

Detecting carcinoma prostate (CaP) after intravesical Bacillus Calmette Guerin (BCG) immunotherapy for non‐muscle invasive bladder cancer (NMIBC) poses diagnostic challenges. Granulomatous prostatitis (GP) has an incidence of 0.8%–3.3% in post‐intravesical BCG patients and 6% incidence in a PIRADS 5 lesion on multiparametric MRI (mpMRI). Patients with GP after intravesical BCG may have clinical, biochemical, and radiological features similar to CaP. In our study, we evaluate the reliability of mpMRI in diagnosing CaP after intravesical BCG therapy.

**Materials and Methods:**

We reviewed the NMIBC patients treated with intravesical BCG therapy between 2017 and 2023 and investigated those who underwent mpMRI and MR fusion biopsy in suspicion of CaP. A total of 120 patients had intravesical BCG immunotherapy, and 10 patients met our selection criteria. We performed a descriptive analysis of these patients and assessed the sensitivity and specificity of mpMRI in diagnosing CaP.

**Results:**

The sensitivity of mpMRI in detecting CaP was 100%, and the specificity was 28.6%. Similarly, the negative predictive value for detecting CaP was 100%, and the positive predictive value was 37.5%. Among patients evaluated with mpMRI, a PIRADS 4 or 5 lesion was seen in 8 (80%) patients, and there was no lesion in 2 (20%) patients. The mpMRI detected 1 lesion in 6 patients (60%) and 2 (20%) in 2 patients. The lesions had a PIRADS score of 4 and 5 in 6 (60%) and 2 (20%) patients, respectively. Among these lesions, 8 (80%) were in the peripheral zone and 2 (20%) in the transition zone. In the MR fusion biopsy of these 10 patients, 7 (70%) had granulomatous prostatitis, and 3 (30%) had CaP.

**Conclusion:**

In our study on evaluating the reliability of mpMRI in diagnosing CaP among post‐intravesical BCG patients, we noted that although PIRADS in mpMRI had high sensitivity in identifying prostate lesions, its specificity for detecting CaP is limited.

## 1 INTRODUCTION

Bladder cancer is the sixth most common cancer in the United States, with non‐muscle invasive bladder cancer (NMIBC) accounting for approximately 75% of the newly diagnosed cases. The primary treatment for intermediate‐ and high‐risk NMIBC includes intravesical immunotherapy with Bacillus Calmette Guerin (BCG) following transurethral resection of bladder tumour (TURBT).^1^ Intravesical immunotherapy helps in reducing the chances of tumour recurrence but is associated with both local and systemic side effects.

Local side effects following intravesical BCG immunotherapy are usually mild and mainly include chemical cystitis, urinary frequency, and hematuria, and it can occur in approximately 62.8% of patients.^2^ One of the rare complications following intravesical instillation of BCG is granulomatous prostatitis (GP), a benign inflammatory condition characterized by granulomas, an aggregate of macrophages surrounded by leukocytes and plasma cells. In post‐intravesical BCG patients, GP happens secondary to the BCG‐stained urine refluxing through the prostatic ducts.^3^ GP has an incidence of 0.8%–3.3% after intravesical BCG.^4^, ^5^ They may have clinical features of an indurated prostate with elevated prostate‐specific antigen (PSA) resembling carcinoma prostate (CaP). These patients warrant further evaluation with radiological imaging.

Multiparametric MRI (mpMRI) is a non‐invasive imaging technique to evaluate CaP. The characteristic features of CaP in mpMRI are a low T2 signal intensity, restricted diffusion area, low apparent diffusion coefficient, and contrast enhancement. Based on these features, the Prostate Imaging – Reporting and Data System (PIRADS), a 5‐point tool, is used to assess the probability of CaP.^6^ GP following intravesical instillation of BCG can have mpMRI features similar to CaP presenting as a PIRADS 4 or 5 lesion. Reports have shown that in patients evaluated with mpMRI for suspicion of CaP, among those with PIRADS 5, the incidence of GP is 6%.^7^ Therefore, diagnosing CaP among patients treated with intravesical BCG immunotherapy is challenging. In our study, we assess the reliability of mpMRI in diagnosing carcinoma prostate after intravesical‐BCG immunotherapy.

## 2 METHODS

This retrospective study was conducted at the Miami Cancer Institute between 2017 and 2023. We queried our hospital database for NMIBC patients who had received intravesical BCG immunotherapy following TURBT. This study was of a retrospective nature, and as the patients were deidentified and were not directly involved, the study met the requirement of waiver of HIPPA authorization, and the institutional review board approval was exempted by Miami Cancer institute‐IRB in June 2023. The BCG‐immunotherapy course had an induction phase with 6 cycles and a maintenance phase with 6 cycles of 3 instillations every 4 to 6 months for 2–3 years. During this course, patients with elevated Serum PSA were investigated radiologically, and those with characteristics suspicious of CaP were further evaluated with MR fusion biopsy. Patients were subjected to mpMRI around 4 to 6 months following the preceding BCG‐immunotherapy. The Prostate Imaging – Reporting and Data System was utilized to grade lesions in the prostate on a scale from 0 to 5. mpMRI positive was defined as those with PIRADS 4 or 5, and mpMRI negative as those with PIRADS ≤3. Later, patients underwent MR‐guided systematic plus targeted biopsy through a transrectal approach. Biopsy positive was defined as those with carcinoma prostate and biopsy negative as those with granulomatous prostatitis.

A total of 120 patients have received intravesical BCG immunotherapy following TURBT for intermediate—and high‐risk NMIBC. Among them, 10 patients had elevated PSA and a mpMRI with MR fusion biopsy. Patients on follow‐up were monitored with repeat PSA every 3 or 6 months, mpMRI, and MR fusion biopsy if required. A descriptive analysis of these patients was performed, and we evaluated the sensitivity and specificity of mpMRI in diagnosing carcinoma prostate.

## 3 RESULTS

**FIGURE 1:**
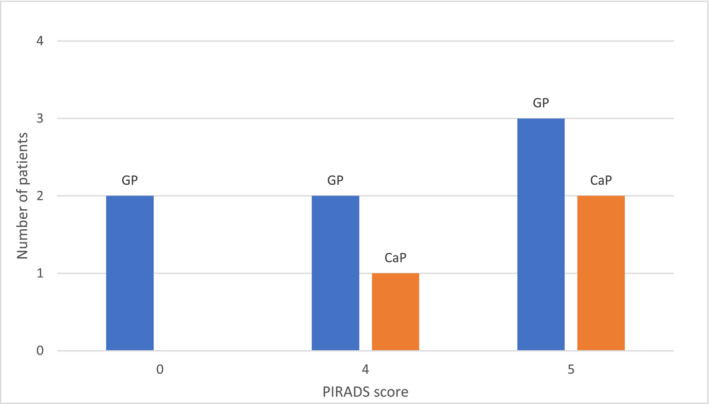
Distribution of granulomatous prostatitis and carcinoma prostate based on PIRADS score.

The sensitivity of mpMRI in detecting PIRADS 4 or 5 lesions in the prostate was 100%, while the specificity was 28.6%. Similarly, although the negative predictive value for detecting CaP was 100%, the positive predictive value was 37.5%. In our series, all 10 patients had completed the induction phase, and 3 (30%) patients had completed the maintenance phase. Among those who did not complete the maintenance phase, 2 (20%) had 5 cycles, 4 (40%) had ≤3 cycles, and 1 (10%) is currently undergoing treatment. During the treatment course, the PSA ranged between 4 and 10 ng/dL in 8 (80%) patients and >10 ng/dL in 2 (20%) patients. On further evaluation with mpMRI, PIRADS 4 or 5 was seen in 8 (80%) patients (figure 1) and no lesion in 2 (20%) patients. Among those with lesions, 6 (60%) had one lesion, and 2 (20%) had two lesions. The distribution of the 10 lesions in mpMRI showed that 8 (80%) were in the peripheral zone (PZ) and 2 (20%) were in the transition zone (TZ) (Table 1).

**TABLE 1:** Biochemical and radiological characteristics of granulomatous prostatitis and carcinoma prostate.

S. no.	PSA (ng/dL)	Digital examination	No. of lesions	Zone of lesion	PIRADS score	MR fusion biopsy	Follow‐up PSA (ng/dL)	Follow‐up MRI	Repeat biopsy
1	6.07	NAD	2	Left posterior PZ Right anterior TZ	4 4	CAP 3 + 4	<0.01	‐	‐
2	16.2	NAD	1	Left anterolateral PZ	5	CAP 3 + 3	<0.01	‐	‐
3	11.23	NAD	2	Left posterior PZ Anterior TZ	4 5	CAP 3 + 4	<0.01	‐	‐
4	10.5	NAD	0	No lesion	0	GP	4.9	No lesion	GP
5	6.1	NAD	0	No lesion	0	GP	‐	‐	‐
6	4.5	NAD	1	Right PZ	5	GP	‐	‐	‐
7	6.5	NAD	1	Left apical medial PZ	4	GP	5.8	No lesion	GP
8	8.3	NAD	1	Left posterior PZ	5	GP	5.2	‐	‐
9	7	NAD	1	Right posterior PZ	5	GP	2.3	‐	‐
10	5.4	NAD	1	Right posterior PZ	5	GP	‐	‐	

Abbreviations: CAP, carcinoma prostate; GP, granulomatous prostatitis; MR, magnetic resonance; MRI, magnetic resonance imaging; NAD, no abnormalities detected; PSA, prostate specific antigen; PZ, peripheral zone; TZ, transitional zone.

**FIGURE 2:**
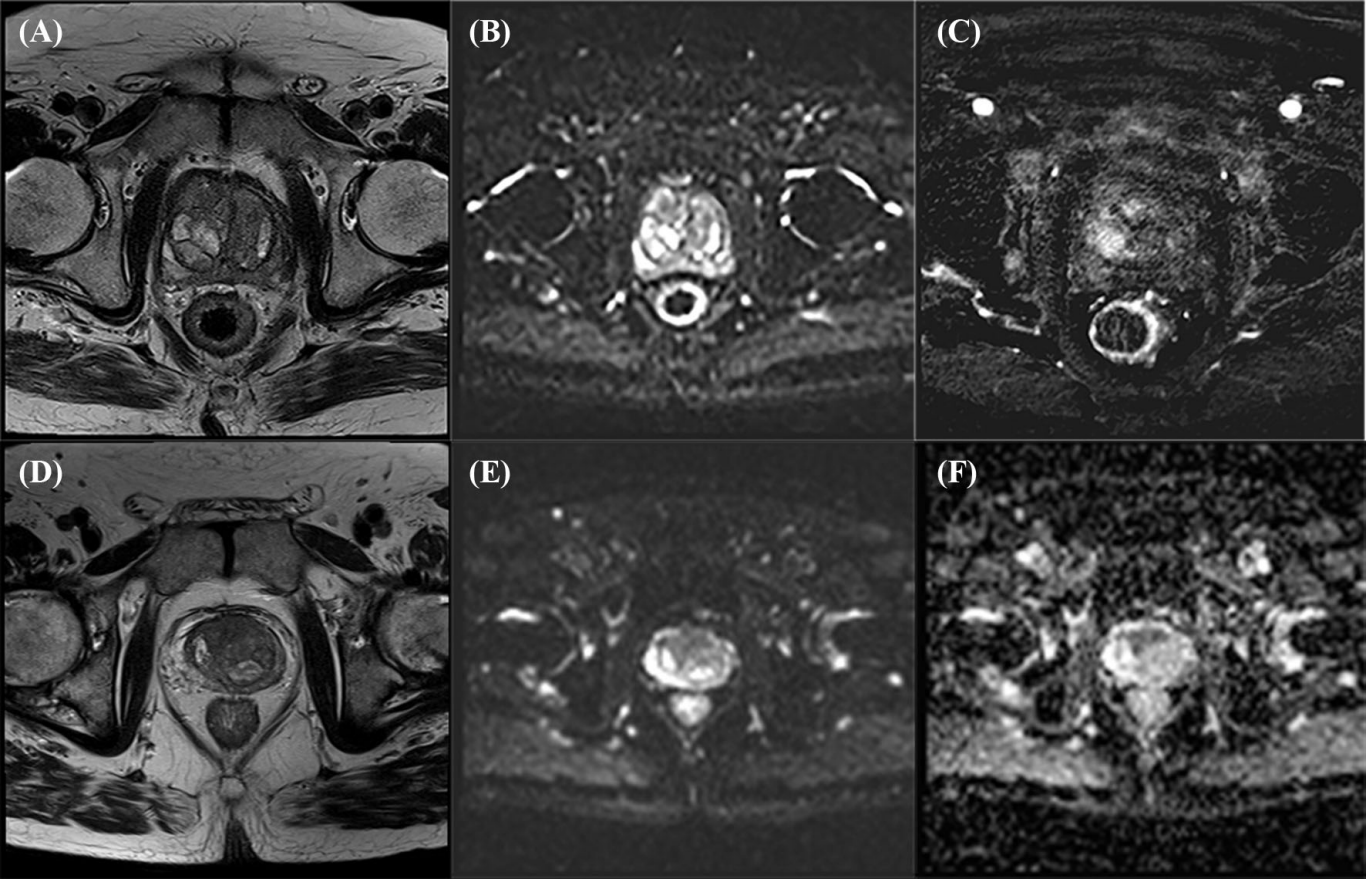
mpMRI images of granulomatous prostatitis and carcinoma prostate showing T2 weighed, diffusion weighed, and dynamic contrast enhancement imaging mpMRI of a post‐intravesical BCG patient with granulomatous prostatitis in axial images showing a 3.6 × 0.8 cm lesion located in the right mid and lower gland extending to the right apex of the prostate in (A) T2 weighted, (B) diffusion‐weighted, and (C) dynamic contrast imaging; mpMRI of a post‐intravesical BCG patient with carcinoma prostate in axial images showing a 1.5 cm lesion located in the left posterior and postero‐lateral peripheral zone of the prostate in (D) T2 weighted, (E) diffusion‐weighted, and (F) dynamic contrast imaging.

In the MR fusion biopsy, 7 (70%) patients were diagnosed with GP, while 3 (30%) were found to have carcinoma prostate (Figure 1). Among those with carcinoma prostate, the mpMRI (Figure 2) showed the lesions were located in the PZ, and the MR fusion biopsy showed 1 patient had a Gleason score of 3 + 3 = 6, and 2 had a score of 3 + 4 = 7. Patients with adenocarcinoma Gleason score 7 underwent robot‐assisted radical prostatectomy (RARP), and the postsurgical specimens were consistent with the same, and one patient underwent radiation therapy. Among those who underwent RARP, there was no intraoperative difficulty or unusual blood loss. Patients with GP were followed up with serial PSA; among them, 3 were recently diagnosed. Follow‐up PSA showed 1 (14.3%) patient had PSA < 4 ng/dL after 6 months, and 3 (42.8%) patients had persistently elevated PSA. Follow‐up mpMRI after 6 months for 2 patients showed no lesions. MR fusion biopsy was repeated for 2 (20%) patients; both had granulomatous prostatitis. Totally 8 (80%) patients are currently under regular follow‐up.

## 4 DISCUSSION

In our study on post‐intravesical BCG therapy patients, we observed that although mpMRI had a high sensitivity for detecting prostate lesions with PIRADS 4 or 5, its specificity for diagnosing CaP was limited. Similarly, though the positive predictive value for detecting CaP was high, the negative predictive value was limited. Therefore, PIRADS alone in mpMRI may not be reliable in diagnosing CaP in this subset of patients. Also, we noted PSA was persistently high in 3/7 (42.9%) patients with GP after 6 months. In 2 patients with persistently elevated PSA on follow‐up, mpMRI showed no lesion, and repeat biopsy was consistent with GP.

In a study by Pepe et al. on 105 patients with PIRADS 5 lesions, they observed 89 (84.5%) had CaP and 16 (15.5%) had benign pathology, of which 6 (37.5%) were GP. In patients with benign pathology, there was a decrease in PSA and downgrading of PIRADS to ≤3 after 6 months, which helped in avoiding a repeat prostate biopsy.^7^ In our series on patients treated with intravesical BCG immunotherapy for NMIBC, 3 of 7 (42.9%) patients with GP on biopsy had persistently elevated PSA on follow‐up, and mpMRI in 2 patients showed no lesions. Though the lesion disappeared, a biopsy was required due to elevated PSA. In an epidemiological study by Tora et al., among 3651 prostate specimens, 39 (1.06%) had GP, and 14 (35.9%) had concomitant CaP.^8^ In our study on mpMRI in post‐intravesical BCG patients, 7 (70%) had GP, and 3 (30%) had CaP.

Ahmed et al observed that in patients with a clinical suspicion of prostate cancer, mpMRI had a sensitivity of 93% and a specificity of 41%.^9^ In our series on patients with post‐intravesical BCG, we observed that though the sensitivity was 100%, the specificity was 28.6%. Hence, mpMRI with PIRADS >3 in this subset of patients may not be suggestive of CaP. Westphalen et al. in their study, observed that 22.8% and 59.2% of patients with a PIRADS 4 and 5 lesion had CaP.^10^ Similarly, Sheridan et al. noted that 18/98 (18%) lesions with PIRADS 5 had benign disease, of which 5/18 (28%) were due to inflammation.^11^ In our series, among patients with PIRADS >3, 3 (37.5%) and 5 (62.5%) had CaP and GP, respectively.

Elena et al., in their study, noted that 11 (1.9%) of 563 cases had GP, and among the 11 cases, 4 (36.4%) had received BCG. In the patients with GP, the mean PSA was 8.74 *±* 6.7 (1.5 to 17.29) ng/mL. MRI showed 8 lesions with sizes ranging between 10 and 35 mm, with 7 (63.6%) in the PZ, 1 (9.1%) in the TZ, and 3 (27.2%) in both PZ and TZ.^12^ Butel et al. examined the zone of involvement of GP in 27 patients following intravesical BCG and observed that 21 (95.5%) patients had involvement of the PZ, 1 (4.5%) had TZ, and 3 (13.6%) had both PZ and TZ. They stated that the obtuse angle of the ducts in the PZ could make it more prone to reflux and inflammation.^13^ In our series, we observed that lesions for GP and CaP in mpMRI were predominantly located in the PZ, 8 (80%) in PZ, and 2 (20%) in TZ.

Our study had several limitations. The rarity of the disease is a significant limitation in our study. The small study population is a direct result of the rarity of the disease in this setting, and there are limited case reports and studies in the literature that investigate this and have similar study populations. Follow‐up of these patients with pre‐and post‐treatment mpMRI may help us to understand the changes in the mpMRI characteristics of these inflammatory lesions. Validating other mpMRI parameters like apparent diffusion coefficient (ADC) and b‐value in diffusion‐weighted imaging among post‐intravesical BCG patients may help in the precise diagnosis of CaP in lesions with PIRADS >3. Currently, MR fusion biopsy is conclusive to rule out CaP in this setting. Future studies incorporating additional radiological tools and parameters to differentiate CaP from GP in post‐intravesical BCG patients can assist in identifying the patients in whom biopsy is mandatory.

## 5 CONCLUSION

In our study on evaluating the reliability of mpMRI in diagnosing CaP following intravesical BCG for NMIBC, we noted that although PIRADS in mpMRI had high sensitivity in identifying prostate lesions with PIRADS 4 and 5, its specificity for detecting CaP was limited. Therefore, PIRADS alone in mpMRI may not offer reliable diagnostic accuracy in this subset of patients. In order to improve the precision in diagnosing CaP, additional radiological tools and parameters may be required.

## AUTHOR CONTRIBUTIONS

*Conception and design*: Murugesan Manoharan and Arjun Pon Avudaiappan. *Administrative support*: Murugesan Manoharan, Ahmed Eldefrawy, and Andrew Renshaw. *Collection and assembly of data*: Pushan Prabhakar and Arjun Pon Avudaiappan. *Data analysis and interpretation*: Pushan Prabhakar and Arjun Pon Avudaiappan. *Manuscript writing*: Arjun Pon Avudaiappan, Pushan Prabhakar, and Rachel Siretskiy. *Final approval of manuscript*: All authors.

## CONFLICT OF INTEREST STATEMENT

Authors have no conflicts of interest to declare.
